# Effects of timing and size of meals prior to farrowing on sow and litter performance

**DOI:** 10.1093/tas/txaa066

**Published:** 2020-05-16

**Authors:** Kiah M Gourley, Analicia J Swanson, Rafe Q Royall, Joel M DeRouchey, Mike D Tokach, Steve S Dritz, Robert D Goodband, Chad W Hastad, Jason C Woodworth

**Affiliations:** 1 Department of Animal Sciences and Industry, College of Agriculture, Kansas State University, Manhattan; 2 Department of Diagnostic Medicine/Pathobiology, College of Veterinary Medicine, Kansas State University, Manhattan; 3 New Fashion Pork, Jackson, MN

**Keywords:** farrowing duration, lactation, piglet performance, survival, transition sow

## Abstract

A total of 727 mixed parity (*µ* = 3.8) sows were used to evaluate the effects of timing and size of meals before farrowing on sow and litter performance. Upon entry to the farrowing house (day 113), sows were blocked by weight within parity and allotted to one of three three feeding management strategies until farrowing: (1) 2.7 kg lactation diet (1.15% standardized ileal digestible lysine and 2,153 kcal/kg net energy) once daily at 0700 hours; (2) four daily meals of 0.67 kg (0100, 0700, 1300, and 1900 hours); (3) ad libitum lactation diet and encouraged to consume feed at 0100, 0700, 1300, and 1900 hours. After farrowing, all sows were provided lactation diets fed on an ad libitum basis until weaning. Data were analyzed for treatment effects within parity category in a mixed model with block as a random effect. Feeding sows ad libitum before farrowing tended to reduce sow body weight (BW) loss (*P* = 0.077) and reduce backfat (BF) loss (*P* = 0.003) from entry into the farrowing house until weaning compared with sows fed four daily meals, with sows fed once daily intermediate. Litter gain from 24 h to weaning tended to be greater (*P* = 0.073) in sows fed on an ad libitum basis or four times daily prior to farrowing compared with sows fed one meal. Piglet weaning weight increased (*P* = 0.050) in sows fed on an ad libitum basis before farrowing, compared with those fed one meal, with those fed four times daily intermediate. There was no evidence for difference in farrowing duration, stillborn rate, colostrum yield, or 24 h piglet survival regardless of treatment. However, from 24 h after farrowing to weaning, sows fed one daily meal prior to farrowing had an increased (*P* = 0.012) percentage of fall-behind pigs compared with sows fed on an ad libitum basis, and increased (*P* = 0.027) preweaning mortality compared with sows fed four daily meals, resulting in reduced (*P* = 0.006) weaned percentage compared with sows fed four daily meals. There was no evidence for difference (*P* > 0.10) in subsequent reproductive performance regardless of treatment. In conclusion, when sows were fed on an ad libitum basis from 2 to 3 d, before farrowing there was an observed improvement in sow BW and BF maintenance during lactation, and piglet weaning weight during lactation. Increased frequency of meals prior to farrowing improved the survival of pigs to weaning compared with sows fed a single meal prior to farrowing.

## INTRODUCTION

The time from initiation to completion of parturition has naturally increased as genetic selection increased litter size by 0.5 pigs from 2012 to 2017 (Stalder, 2018). Longer farrowing durations can have negative effects on sow health and survival of piglets during parturition and lactation. Increased farrowing duration has been associated with a greater number of stillbirths (Oliviero et al., 2010; Feyera et al., 2018). This is likely due to long farrowing process causing hypoxia of piglets resulting in stillbirths, or negatively impacting live born piglet growth and survivability beyond the first few hours of life (Herpin et al., 1996). It remains unknown if increased farrowing duration is caused by stillborn piglets blocking the birth canal, if the sow has depleted her energy stores during parturition and slows contractions, or if other genetic and environmental factors are resulting in increased farrowing duration (Vanderhaeghe et al., 2013).

Recent data from a commercial sow study demonstrated that on average, parturition lasts for 4 h, but can range from 30 min up to 12 h to complete (Gourley et al., 2019). Feyera et al. (2018) conducted a retrospective analysis to evaluate the timing of the last meal prior to parturition on farrowing duration and stillborn rate. They concluded that when sows had been offered a meal 3 h or less before parturition, she had a shorter farrowing duration, decreased need for farrowing assistance, and reduced stillborn rate, in comparison to sows that had been offered their last meal >6 h prior to farrowing. They hypothesized that this was due to higher plasma glucose levels at the onset of farrowing which resulted in more energy to be readily available during the farrowing process. Oliviero et al. (2009) observed longer farrowing durations were associated with a number of factors including sow housing, sow backfat (BF), sow constipation score, and number of stillborn pigs. Several peripartum feeding strategies have been investigated to reduce farrowing duration and stillborn rate with little to no benefit observed (Vallet et al., 2013; Manu et al., 2018; van den Bosch et al., 2019).

Currently, commercial farms utilize many different feeding management strategies once sows are moved into farrowing crates until the onset of parturition such as feeding one set feed amount in the morning, feeding two smaller meals twice daily, ad libitum, or other combinations. To our knowledge, no previous study has focused specifically on number of meals, and feed availability from day 113 of gestation to parturition, and its effect on farrowing duration and piglet survival. Therefore, the objective of this study was to determine the effect of amount of feed and frequency of feed delivery on the parturition process, sow and litter performance, and survivability of piglets.

## MATERIALS AND METHODS

The Kansas State University Institutional Animal Care and Use Committee approved the protocol used in this experiment. A total of 727 sows (Fast Large white × PIC Landrace) were used at a commercial sow farm in southern Minnesota (New Fashion Pork, Jackson, MN). During gestation, sows were housed in individual stalls. The farrowing house was equipped with individual crates each containing a shelf feeder with a hopper for sows, nipple waterer for sows, and heat mat for piglets. On the day of gestation when sows entered the farrowing house –(day 113 ± 2), sows were weighed and BF was measured at the P2 position (Renco Lean Meter, S.E.C. Repro Inc., Quebec, Canada). At this time, sows were blocked by body weight (BW) within parity category (gilts, parity 1, and parity 2+) and allotted to one of three feeding management strategies. Treatments consisted of (1) sows fed 2.7 kg lactation diet once daily at 0700 hours; (2) sows fed a total of 2.7 kg lactation diet in four daily 0.68 kg meals (0100, 0700, 1300, and 1900 hours); and (3) sows were offered ad libitum lactation diet (ensured at least 2.5 kg of diet in the feeder) and encouraged to consume meals four times daily by making the sows stand (0100, 0700, 1300, and 1900 h). Although sows fed once daily (0700 hours) were not made to stand at the three time points when other sows were fed, naturally the majority would stand at that time due to a sow in an adjacent stall receiving feed. When sows entered the farrowing house (1300 hours), treatments 2 and 3 were fed their first meal, whereas treatment 1 did not receive their first meal in the farrowing house until the following morning. Prior to entry to the farrowing house, sows were fed 2.04 kg of gestation diet in a single daily meal offered at 0700 hours. Diets were formulated to meet or exceed nutrient requirements (Table 1) and were manufactured at the New Fashion Pork feed mill (Estherville, IA). Feed samples were collected twice each week from the feeders at the farm. Samples were pooled and used for chemical analysis.

**Table 1.** Dietary composition^1^

Ingredient, %	Lactation diet
Ground corn	46.30
Dried distillers’ grain with solubles	25.00
Soybean meal, 47.5% CP	21.3
Vegetable oil blend^2^	2.85
Limestone	1.38
Monocalcium phosphate, 21% P	0.93
Liquid energy^3^	0.75
L-Lysine HCl	0.52
Salt	0.28
Vitamin premix^4^	0.25
L-Threonine	0.17
Choline Chloride, 60%	0.10
L-Valine	0.06
L-Methionine	0.05
L-Tryptophan	0.03
Standardized ileal digestible amino acids, %	
Lysine	1.15
Methionine and cysteine:lysine	0.50
Threonine:lysine	0.65
Tryptophan:lysine	0.18
Valine:lysine	0.70
Isoleucine:lysine	0.56
Total lysine, %	1.30
Crude protein, %	20.1
Metabolizable energy, kcal/kg	3,320
Net energy, kcal/kg	2,535
Calcium, %	0.77
Phosphorus, %	0.66
Analyzed composition^5^, %	
Dry matter	89.5
Crude protein	20.6
Calcium	1.06
Phosphorus	0.67

^1^Lactation diets were fed upon entry to farrowing house, according to treatment feeding strategy. After farrowing, sows received lactation diet ad libitum until weaning.

^2^Build R2 (Feed Energy Company, Pleasant Hill, IA).

^3^XFE Liquid Energy; alcohol-based liquid product (XFE Products, Des Moines, IA).

^4^Provided per kg diet: 10,409 IU vitamin A; 447 IU vitamin D_3_; 36.3 µg vitamin D; 70 IU vitamin E; 250 mg vitamin C; 3.7 mg vitamin K; 41.4 mg niacin; 27.5 mg pantothenic acid; 1.7 mg folic acid; 2.1 mg thiamine; 8.1 mg Riboflavin; 4 mg Pyridoxine; 35.4 mg vitamin B_12_; 0.4 mg Biotin; 221 mg Fe from Fe sulfate; 0.3 mg Se from Na selenite; 18.5 mg Cu from Intelibond C; 132 mg Zn from Intelibond Z; 33 mg Mn from Mn oxide; 1.2 I from calcium iodate; 0.44 mg Cr from Cr propionate; 500 FTU phytase.

^5^A total of eight samples (1 per week) of lactation diet from within the weekly pooled samples were analyzed in duplicate at a commercial laboratory (Ward Laboratories, Kearney, NE).

All feeding strategies were administered via hand feeding from a feed cart equipped with a scale until the start of parturition. At the start of parturition, feed remaining in the feeder was weighed to calculate total feed consumed from entry to the farrowing house until parturition. Sows were not fed during parturition; however, upon completion, sows were fed lactation diet ad libitum until weaning. Pre-farrow feed intake was recorded for all individual sows and on a subsample of 310 sows during lactation.

During parturition, sows were continuously monitored for 24 h. When a piglet was born, time was recorded, pigs were dried off using a desiccant (Tech dry; Techmix LLC., Stewart, MN) and paper towels. Umbilical cords were then tied and cut to approximately 10 cm in length. Additionally, pigs were given an individual ear tag for identification and weighed before placing them next to the sow. Stillborn and mummified fetuses were also weighed and birth time recorded. During parturition, farrowing assistance was provided after 30 to 45 min with no farrowing progress evidence from the time a previous was pig born. When provided, farrowing assistance was noted on the litter record. The farrowing process was complete when no new pig had been born after 1 h and placenta expulsion observed. At 24 h after birth of the first piglet in each litter, piglets were individually weighed to calculate colostrum intake and colostrum yield. All piglets remained with their birth sow during the trial until weaning.

Sow blood glucose was measured on a subsample of 345 sows. Blood was obtained by pricking the ear auricular vein with a needle, and using a Glucometer (AimStrip Plus, Germaine Laboratories Inc., San Antonio, TX) to measure blood glucose. Measurements were collected at three time points during parturition: start of farrowing, 2 h after start of farrowing, and at the end of farrowing.

On day 3 after birth, all piglets received 1cc iron (Uniferon Iron Dextran 200 mg/mL; Pharmacos Inc.; Watchung, NJ), 2 cc haemophilis parasuis vaccine (Cambridge Technologies; Worthington, MN) and males were castrated. All piglet mortalities prior to 24 h were recorded and classified as either (1) died at birth (died within an hour of birth) or (2) laid on (due to crushing by the sow). Due to the health status of the farm, no cross-fostering occurred, and no nurse sows were utilized. At 24 h, piglets <600 g were identified and euthanized according to farm protocol. Fall-behind pigs and mortalities were weighed, and date was recorded for all litters from birth to weaning. Fall-behind piglets were classified as losing weight for multiple days during lactation or sustaining a life-threatening injury; they were removed from the sow and humanely euthanized (Euthanex AgPro; Nutriquest; Mason City, IA). Piglets were not provided with creep feed during lactation.

On the day prior to weaning, all piglets were individually weighed to measure litter growth and litter weight CV. On the day of weaning (day 21± 3), sows were weighed, and BF was measured at the P2 position. Sows were moved to individual gestation stalls and checked once daily for signs of estrus using a boar and back pressure test for 42 d postfarrowing. Wean to first service interval and day 30 conception rate were collected on a total of 562 sows that remained after culling due to age (*n* = 160), injury (*n* = 3), or infertility (*n* = 2). Farrowing rate, subsequent total born, born alive, and stillborn were collected by farm employees and accessed through the farm database (PigCHAMP; Ames, IA).

### Calculations

Sow BW post-farrowing was calculated by subtracting the weight of conceptus from sow BW at day 113. Weight of conceptus was calculated for each sow using the equations described by Thomas et al. (2018), using the variables: total born, average piglet birth weight, and gestation length. Piglet birth weight CV, 24 h weights and weaning weights were calculated from using individual pig weights within each litter at each time point.

Colostrum intake for individual piglets was calculated using the equation developed by Theil et al. (2014a), using piglet 24 h weight gain, suckling duration, and birth weight. Colostrum yield for each sow was calculated as the sum of the colostrum intake of the pigs in the litter. If a piglet died before 24 h, the assumption was that there was no colostrum intake by that piglet.

Time from loading into farrowing crates to the onset of farrowing was calculated as (date of farrowing – date of entry to farrowing house), in days. Time-consuming meals prior to farrowing was calculated as (start time of parturition − time the first meal was delivered upon entry to the farrowing house), in hours. Time since last meal was calculated as (farrow start time – time of last meal), in minutes. Pig to teat ratio for each sow was calculated as number of born alive piglets divided by functional teats. Pre-farrow feed intake is the sum of feed consumed from entry to the farrowing house until the start of parturition. Total feed intake is total pre-farrow feed intake plus total lactation feed intake. Farrowing duration was calculated as the time from birth of first pig to birth of last pig. Average birth interval per sow was calculated as the actual interval between each piglet and then averaged for the sow. Percentage assisted was calculated by dividing the number of pigs sleeved per litter by the number of total born pigs per litter.

### Chemical Analysis

A total of eight samples (1 per week) of lactation diet from within the weekly pooled samples were sent to a commercial laboratory (Table 1; Ward Laboratories, Kearney, NE). Samples were analyzed in duplicate for crude protein (AOAC 990.03, 2006), Ca (Campbell and Plank, 1991; Kovar, 2003), and P (Campbell and Plank, 1991; Kovar, 2003).

### Statistical Analysis

Data were analyzed using generalized linear mixed models where dietary treatment within parity category was a fixed effect, with random effect of block. Heterogeneous variance by treatment was tested for each variable and used if it significantly improved the model fit.

Sow BW, BF depth, litter weights, mean piglet BW, litter gain, colostrum yield and intake, piglet BW CV, total born, litter counts, and feed intake were fit using a normal distribution. Farrowing duration and birth interval were log transformed to normalize data and then fit using a normal distribution. Wean to estrus interval was fit using a negative binomial distribution. Percentage born alive, stillborn, assisted, survived to 24 h, fall-behind, mortality, weaned, in estrus by day 7, conception rate and farrowing rate were fit using a binomial distribution.

Covariates were used if they significantly improved the model fit. Residuals and the Bayesian Information Criterion were used as an indication of improved model fit. Entry weight was used as a covariate for sow weaning weight, sow entry BF, and weight change from entry to weaning. Sow BF at entry was used as a covariate for sow BF at weaning and BF change from entry to weaning. Parity was used as a covariate for total sow feed intake. Total born was used as a covariate for total born litter weight, and total born, born alive and 24 h mean piglet BW, and total born individual pig weight CV. Born alive was used as a covariate for born alive and 24 h litter weights and CV. Both lactation length and born alive were used as a covariate for litter weaning weight and mean piglet weaning weight. Pig-to-teat ratio was used as a covariate for litter gains, colostrum yield, and colostrum intake. Sow blood glucose was analyzed for a time × treatment interaction, and main effects of time and treatment using a repeated measures statement. Statistical models were fit using the Lme function (lmer package of R, version 3.5.2). Results were considered significant at *P* < 0.05 and marginally significant at 0.05 ≤ *P* < 0.10.

## RESULTS

### Timing of Treatments

Time from loading to farrowing was 0.3 d shorter (*P* = 0.005) for sows fed on an ad libitum basis prior to farrowing, compared with sows that received one meal daily (Table 2). Time-consuming meals prior to farrowing (in hours) was shorter (*P* = 0.001) for sows fed 2.7 kg once daily compared with the other two treatments, as would be expected due to feeding strategy design of the trial. As a result, time since last meal in relation to start of parturition decreased (*P* = 0.001) in sows fed 0.68 kg or ad libitum and made to stand every 6 h compared with those fed 2.7 kg once daily. Total pre-farrow feed intake was increased (*P* = 0.001) in sows fed on an ad libitum basis compared with the other feeding strategies. These results validated that the feeding strategies had been applied successfully, in order to create differences in timing of meals and amount of feed provided prior to parturition.

**Table 2.** Timing and amount of feed delivered to sows prior to farrowing on sow performance^1^

Response	2.7 kg × 1 delivery	0.68 kg × 4 deliveries	Ad libitum × 4 deliveries	SEM	*P*-value
Count, *n*	242	245	240	—	—
Parity, *n*	3.8	3.8	3.9	—	—
Gestation length, day	115.5	115.5	115.4	0.08	0.323
Lactation length^2^, day	21.7	21.7	21.9	0.09	0.043
Time from loading to farrow^3^, day	3.2^a^	3.1^ab^	2.9^b^	0.08	0.005
Time consuming meals prior to farrow^4^, h	57.4^b^	70.9^a^	65.1^a^	1.94	0.001
Time from last meal to farrrowing^5^, min	605^a^	196^b^	216^b^	25.6	0.001
Sow BW, kg					
Entry	260.3	259.7	260.2	1.78	0.734
Post-farrow^6^	247.2	245.9	245.9	1.75	0.237
Weaning	237.7	236.1	239.4	1.10	0.077
Sow weight change, kg					
Entry to weaning	−23.8	−25.4	−22.1	1.10	0.077
Post-farrow−weaning	−10.7^ab^	−11.4^b^	−8.4^a^	0.94	0.035
Sow BF, mm					
Entry	13.4	13.8	14.0	0.24	0.179
Weaning	11.6^ab^	11.1^b^	11.8^a^	0.16	0.003
Sow BF change, mm					
Entry to weaning	−2.2^ab^	−2.7^a^	−1.9^b^	0.16	0.003
Sow feed intake					
Total pre−farrow feed intake^7^, kg	7.5^b^	7.9^b^	9.7^a^	<0.31^10^	0.001
Lactation average daily feed intake^8^, kg	4.8	5.0	5.1	0.09	0.175
Total feed intake^9^, kg	116.0^b^	117.8^ab^	123.8^a^	<3.50^11^	0.018

^1^A total of 727 mixed parity sows were used from entry into the farrowing house (day 113 ± 2 of gestation) until weaning. Sows were weighed, blocked by parity category and weight, and allotted to treatment at time of entry to the farrowing house. Treatments consisted of (1) sows fed 2.7 kg lactation diet once daily at 0700 h;( 2) sows fed 2.7 kg lactation diet four times daily in 0.68 kg meals (0100, 0700, 1300, 1900 hours); and 3) sows fed on an ad libitum basis lactation diet and encouraged to consume meals every 4 times daily (0100, 0700, 1300, and 1900 hours). Weaning occurred on d 21.7 (± 3.3 d) of lactation.

^2^Tukey adjustment resulted in no mean separation.

^3^Days spent in farrowing crate prior to parturition = (Farrowing date – load date).

^4^Number of hours sow received treatments prior to farrowing. Sows were loaded into farrowing crates at 1300 h each day, therefore sows consuming 0.68 kg meals and ad libitum received feed at loading. Sows receiving 2.7 kg once a day did not receive feed until the following morning.

^5^Time from last meal delivery to the birth of first pig.

^6^Calculated from equation by Thomas et al (2018).

^7^Sum of feed consumed from loading to farrowing, measured on all sows.

^8^Lactation feed intake was measured on a subsample of 310 sows.

^9^Sum of feed consumed from loading to weaning, measured on a subsample of 310 sows.

^10^Heterogenous variance by treatment, SEM = 0.23, 0.25, and 0.31 for sows receiving 2.7 kg × 1 delivery, 0.68 kg × 4 deliveries, and ad libitum × 4 deliveries, respectively.

^11^Heterogenous variance by treatment, SEM = 3.47, 3.50, and 3.33 for sows receiving 2.7 kg × 1 delivery, 0.68 kg × 4 deliveries, and ad libitum × 4 deliveries, respectively.

### Sow BW, BF, and Feed Intake

There was no evidence sow BW and BF were different (*P* > 0.10) at the start of the trial (entry to the farrowing house). Calculated sow BW post-farrowing was similar (*P* > 0.10), which was expected due to no change in weight of conceptus from dietary treatments applied for 3 d prior to farrowing. Sow BW at weaning was marginally heavier (*P* = 0.077) in sows that had been fed on an ad libitum basis prior to farrowing compared with those fed 0.68 kg every 6 h. As a result, sows that consumed feed ad libitum prior to farrowing had reduced sow BW loss from post-farrow to weaning (*P* = 0.035) and tended to have reduced sow BW loss from entry to weaning (*P* = 0.077) compared with those fed 0.68 kg every 6 h, with sows fed 2.7 kg once daily intermediate. Sow BF loss during lactation was reduced (*P* = 0.003) in sows fed on an ad libitum basis compared to sows fed 0.68 kg every 6 h, resulting in greater (*P* = 0.003) BF at weaning in sows fed on an ad libitum basis prior to farrowing, compared with sows fed 0.68 kg every 6 h, with sows fed 2.7 kg once daily intermediate.

Sow lactation feed intake was numerically increased (*P* = 0.175) in sows fed on an ad libitum basis prior to farrowing (5.1 vs. 4.8 kg/d) compared with sows fed 2.7 kg once daily, with those fed 0.68 kg every 6 h intermediate. Combining lactation feed intake with pre-farrow feed intake resulted in increased (*P* = 0.018) total feed intake per sow for sows fed on an ad libitum basis prior to farrowing compared to sows fed 2.7 kg once daily, with those fed 0.68 kg every 6 h intermediate.

### Farrowing Duration and Piglet Survivability

There was no evidence total born pigs and percentage of pigs born alive or stillborn were different (*P* > 0.10) due to feeding strategy (Table 3). There were differences (*P* < 0.001) in percentage of pigs assisted per sow. The sows fed on an ad libitum basis prior to farrowing, had the highest percentage assistance, followed by sows fed once daily prior to farrowing, with those receiving 0.68 kg every 6 h having the lowest assistance rate. Farrowing duration, birth interval, and time to birth of 6^th^ pig were similar (*P* > 0.05) across feeding strategies (Table 4).

**Table 3.** Timing and amount of feed delivered to sows prior to farrowing on litter performance^1^

Response	2.7 kg × 1 delivery	0.68 kg × 4 deliveries	Ad libitum × 4 deliveries	SEM	*P*−value
Litter characteristics					
Total born, *n*	16.1	15.7	16.0	0.23	0.351
Born alive, %	93.4	93.8	93.6	0.45	0.664
Stillborn, %	6.6	6.1	6.4	0.44	0.667
Assisted, %	16.1^b^	13.7^c^	19.6^a^	1.11	0.001
Litter size at 24 h, *n*	13.9	13.6	13.8	0.20	0.432
Litter size at weaning, *n*	11.2	11.3	11.2	0.15	0.752
Litter weight, kg					
Total born, 0 h	19.4	20.0	19.4	0.20	0.053
Born alive, 0 h	18.2^b^	18.7^a^	18.3^ab^	0.19	0.046
24 h	18.7	19.2	18.9	0.21	0.219
Weaning^2^	53.1	55.2	54.1	0.65	0.083
Mean piglet BW, kg					
Total born, 0 h	1.24	1.28	1.25	0.012	0.055
Born alive, 0 h	1.25^b^	1.29^a^	1.26^ab^	0.013	0.045
24 h	1.36	1.40	1.37	0.012	0.088
Weaning^2^	4.80^b^	4.90^ab^	4.94^a^	0.045	0.050
Litter gain 0 to 24 h, kg	1.38	1.27	1.29	0.048	0.218
Litter gain 24 h to wean, kg	34.08	35.94	35.26	<0.620^6^	0.073
CV of individual pig weights, %					
Total born	23	22	23	0.40	0.218
24 h	23	22	23	0.40	0.143
Weaning^2^	19	19	19	0.40	0.486
Colostrum intake^3^, g/pig	418	422	415	<6.0^7^	0.606
Colostrum yield^4^ kg/sow	5.7	5.7	5.6	0.08	0.471
Pig:teat^5^	0.98	0.97	0.98	0.015	0.832

^1^A total of 727 mixed parity sows were used from entry into the farrowing house (day 113 ± 2 of gestation) until weaning. Sows were weighed, blocked by parity category and weight, and allotted to treatment at time of entry to the farrowing house. Control sows were fed 2.7 kg lactation diet once daily at 0700 hours; sows were fed 2.7 kg lactation diet four times daily in 0.68 kg meals (0100, 0700, 1300, and 1900 hours); sows were fed on an ad libitum basis lactation diet and encouraged to consume meals every four times daily (0100, 0700, 1300, and 1900 hours).

^2^Lactation length averaged 21.7 ± 3.3 d.

^3^Calculated based on equation by Theil et al. (2014a).

^4^Sum of individual colostrum intake for all pigs in the litter.

^5^Pigs per functional teat.

^6^Heterogenous variance by treatment, SEM = 0.620, 0.534, and 0.535 for sows receiving 2.7 kg × 1 delivery, 0.68 kg × 4 deliveries, and ad libitum × 4 deliveries, respectively.

^7^Heterogenous variance by treatment, SEM = 5.3, 6.0, and 5.2 for sows receiving 2.7 kg × 1 delivery, 0.68 kg × 4 deliveries, and ad libitum × 4 deliveries, respectively.

**Table 4.** Timing and amount of feed delivered to sows prior to farrowing on farrowing duration, birth order and survival^1^

Response	2.7 kg × 1 delivery	0.68 kg × 4 deliveries	Ad libitum × 4 deliveries	SEM	*P*−value
Farrowing duration^2^, min	209	200	214	1.16	0.226
Birth interval, min	13.6	13.7	14.3	1.07	0.448
Birth time of sixth pig, min	90.2	93.1	95.5	3.82	0.620
Outcome to 24 h^3^					
Died at birth^4^, %	1.3	1.5	1.3	0.22	0.839
Laid on, %	5.0	5.2	5.2	0.41	0.950
Survived to 24 h, %	93.6	93.3	93.4	0.44	0.912
Outcome to weaning^3^					
Euthanized at 24 h, %	2.5	2.1	2.8	0.31	0.110
Fall−behind, %	7.5^a^	6.3^ab^	5.9^b^	0.52	0.012
Dead, %	7.6^a^	6.1^b^	6.6^ab^	0.49	0.027
Weaned, %	74.3^b^	77.6^a^	76.1^ab^	0.80	0.006

^1^A total of 727 mixed parity sows were used from entry into the farrowing house (day 113 ± 2 of gestation) until weaning. Sows were weighed, blocked by parity category and weight, and allotted to treatment at time of entry to the farrowing house. Treatments consisted of (1) sows fed 2.7 kg lactation diet once daily at 0700 hours; (2) sows fed 2.7 kg lactation diet four times daily in 0.68 kg meals (0100, 0700, 1300, and 1900 hours); and (3) sows fed on an ad libitum basis lactation diet and encouraged to consume meals every four times daily (0100, 0700, 1300, and 1900 h). Weaning occurred on day 21.7 (± 3.3 d) of lactation.

^2^Time from birth of first piglet to last piglet.

^3^Calculations use the count of the variable divided by born alive count. Analyzed as a binominal.

^4^Died within an hour after birth, includes low viable, deformed, and savaged pigs.

Percentage of pigs that died at birth or were laid on within 24 h after birth were similar (*P* > 0.10) across treatments, which resulted in no evidence for difference (*P* > 0.10) in piglet survival to 24 h. Piglets that were euthanized at 24 h due to low birth weight, or injury were similar (*P* = 0.110) across treatments. Percentage of fall behind pigs was reduced (*P* = 0.012) in sows fed on an ad libitum basis prior to farrowing compared with those fed once daily prior to farrowing, with those fed 0.68 kg every 6 h prior to farrowing intermediate. Piglet mortalities from 24 h to weaning were reduced (*P* = 0.027) in sows fed 0.68 kg every 6 h compared with those fed once daily prior to farrowing. Although litter size at weaning was similar across treatments, the total percentage of pigs weaned was increased (*P* < 0.05) in those fed 0.68 kg every 6 h prior to farrowing compared with those restricted to 2.7 kg once daily, with ad libitum fed sows intermediate.

### Litter Performance and Colostrum Production

Feeding strategies prior to farrowing did not influence (*P* = 0.122) total born litter weight. Born alive litter weight was heavier (*P* = 0.046) in sows fed 0.68 kg every 6 h compared with the control, with sows fed on an ad libitum basis intermediate. There was no evidence for difference (*P* > 0.10) in colostrum yield or intake, which resulted in similar 24 h litter weights and litter gain in the first 24 h. Litter weight at weaning and litter gain from 24 h to weaning was marginally increased (*P* < 0.10) in sows fed 0.68 kg every 6 h or ad libitum prior to farrowing compared to those fed 2.7 kg once daily.

Mean piglet BW was marginally greater (*P* = 0.055) in total born pigs and greater (*P* = 0.045) in born alive pigs in sows fed 0.68 kg every 6 h prior to farrowing compared with those fed 2.7 kg once daily, with those fed on an ad libitum basis prior to farrowing intermediate. This resulted in marginally increased (*P* = 0.088) piglet weights at 24 h in sows fed 0.68 kg every 6 h compared with the other two treatments. At weaning, pigs from sows fed on an ad libitum basis prior to farrowing were heavier (*P* = 0.050) compared with sows fed 2.7 kg once daily prior to farrowing, with those fed 0.68 kg every 6 h intermediate. There was no evidence for difference (*P* > 0.10) in CV for piglet BW at birth, 24 h, or weaning, regardless of feeding strategy.

### Sow Blood Glucose and Subsequent Reproductive Performance

**Figure 1.**
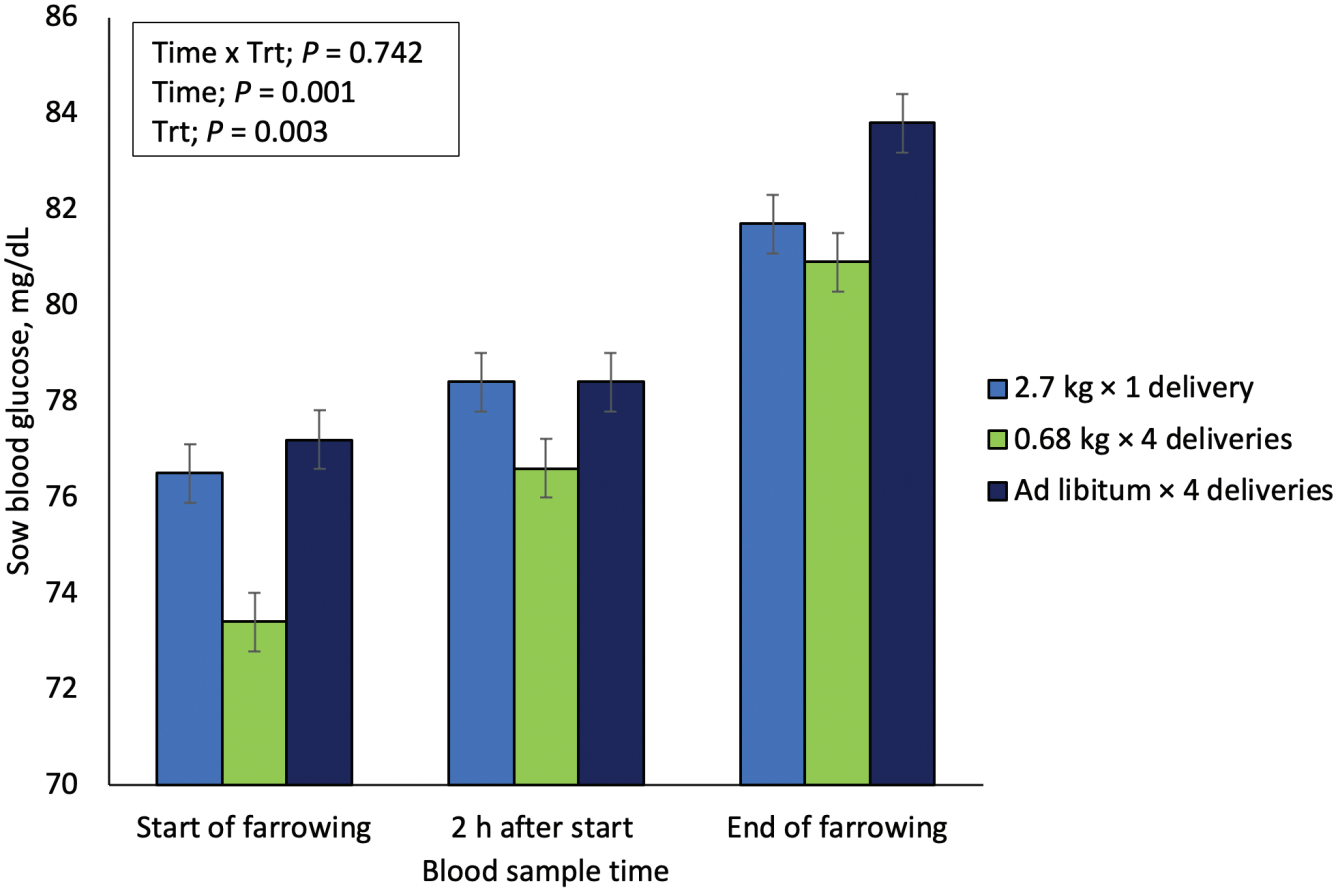
A subsample of 345 mixed parity sows were used for blood glucose measurement. Blood glucose was measured by pricking the ear vein and using a glucometer to read the glucose concentration (mg/dL) at three time points during parturition. End of farrowing was characterized as no new piglets for 45 minutes and evidence of placenta expulsion.

There was no evidence for a treatment × time point interaction in sow blood glucose (Figure 1). A feeding strategy main effect was observed where sows fed on an ad libitum basis had the greatest (*P* = 0.003) blood glucose concentrations (*µ* = 79.8 mg/dL) at each time point (start of farrowing, 2 h after start of farrowing, and at the end of farrowing), sows fed 0.68 kg every 6 h had the lowest blood glucose concentrations (*µ* = 76.9 mg/dL), with sows fed 2.7 kg once daily intermediate (*µ* = 78.9 mg/dL). Additionally, a main effect of time was observed, where blood glucose concentrations increased (*P* = 0.001) from the start of farrowing (*µ* = 75.7mg/dL), to 2 h after the onset of parturition (*µ* = 76.9 mg/dL) and were the highest at the end of farrowing (*µ* = 79.8 mg/dL).

After culling at weaning, a total of 562 females remained in the herd and were used to measure subsequent reproductive performance (Table 5). There was no evidence for difference (*P* > 0.10) in wean-to-estrus interval, percentage of females in estrous by day 7 or 20 after weaning, conception rate, or farrowing rate regardless of feeding strategy prior to farrowing. Subsequent total born, born alive, and stillborn were similar (*P* > 0.10) across all pre-farrow feeding strategies.

**Table 5.** Timing and amount of feed delivered to sows prior to farrowing on subsequent reproductive performance^1^

Response	2.7 kg × 1 delivery	0.68 kg × 4 deliveries	Ad libitum × 4 deliveries	SEM	*P*−value
Count, *n*	188	188	186	−−	−−
Wean to estrus interval^2^, day	4.6	4.0	4.3	<0.182^3^	0.162
Estrous by day 7, %	95.7	96.3	96.2	1.47	0.958
Estrous by day 20, %	97.3	99.4	98.9	1.17	0.204
Conception rate^4^, %	89.4	88.3	86.6	2.51	0.703
Farrowing rate^5^, %	85.1	87.8	85.0	2.62	0.673
Subsequent litter					
Count^6^, *n*	160	165	158	−−	−−
Total born, *n*	13.7	14.0	13.7	0.26	0.492
Born alive, %	93.4	93.3	92.8	0.55	0.809
Stillborn, %	6.6	6.7	7.2	0.59	0.809

^1^A total of 562 mixed parity sows remaining in the herd were used to collect subsequent reproductive performance. Sows were culled after lactation due to old age (*n* = 160), injury (*n* = 3), or infertile (*n* = 2).

^2^Sows were monitored for 42 d after weaning for signs of estrus.

^3^Heterogenous variance by treatment, SEM = 0.18, 0.17, and 0.17 for sows receiving 2.7 kg × 1 delivery, 0.68 kg × 4 deliveries, and ad libitum × 4 deliveries, respectively.

^4^Sows confirmed pregnant at day 30 divided by total number bred.

^5^Sows farrowed divided by total number bred.

^6^Sows that farrowed a subsequent litter.

## DISCUSSION

### Farrowing Duration, Stillborn Rate, and Farrowing Assistance

In 2017, the average percentage of stillbirths and mummified fetuses in U.S. sow herds was 9.8% (Stalder, 2018). It has been observed that longer farrowing duration is associated with increased piglet asphyxia and stillborn rate (Langendijk and Plush, 2019). There was no evidence for difference in stillborn rate or farrowing duration in the current study, regardless of feeding amount or meal frequency prior to farrowing. In contrast, Feyera et al. (2018) observed an increase in the probability of stillbirths when the time from last meal to parturition exceeded 6 h compared with those that had received feed <6 h before farrowing. These different results may be due to the difference in average farrowing duration, farrowing assistance, total born or dietary energy supplied in the pre-farrow period between studies. In the present study, farrowing duration averaged 3.5 h, which is much shorter than the average 5.8 h observed by Feyera et al. (2018). Total born was also lower in the present study (16.0 vs. 17.1 pigs) compared with Feyera et al. (2018). We speculate that the lower total born in the present study resulted in a shorter mean farrowing duration, therefore limiting the number of sows experiencing farrowing fatigue due to pre-farrow fasting. Farrowing assistance was more frequent in the present study which may have reduced the differences in stillbirths between treatments, whereas Feyera et al. (2018) allowed up to a 60 min birth interval before intervention. Additionally, sows in the current study would have received 8.9 to 11.0 Mcal/d metabolizable energy (ME) prior to farrowing compared with only 7.4 Mcal/d ME provided the last 3 d prior to farrowing by Feyera et al. (2018). The increased dietary energy provided in the current study pre-farrow may have reduced the impact of time from last meal prior to farrowing on farrowing duration, as previously observed (Feyera et al., 2018).

Interestingly, the percentage of pigs assisted was highest in sows fed on an ad libitum basis prior to farrowing, and lowest in sows fed small meals every 6 h prior to farrowing. This result would suggest that increased frequency and a smaller meal size prior to farrowing had a positive impact on the sow’s ability to expel piglets without assistance, which would be similar to observations by Feyera et al. (2018). Although sows fed on an ad libitum basis were encouraged to consume a meal every 6 h prior to farrowing and had 24 h access to feed, perhaps they ate fewer large meals and had not consumed a meal as frequently as those that were restricted to 0.68 kg every 6 h. Indeed, Guillemet et al. (2010) noted that sows prefer to work for food compared with ad libitum access, which demonstrates a change in feeding behavior when sows receive free access to feed.

### Piglet Survivability and Colostrum Production

Our current study demonstrated that feeding frequency and feed quantity had little observable impact on piglet survival in the first 24 h since the percentage of pigs that were crushed or died at birth were similar across treatments. The improvement in piglet survival became evident from 24 h to weaning as the number of mortalities was reduced in sows fed every 6 h prior to farrowing compared with sows fed one daily meal prior to farrowing. This could be explained in part by the heavier average piglet birth weight (1.29 vs. 1.24 kg) improving survival to weaning, which is well established (Baxter et al., 2008; Oliviero et al., 2019). Furthermore, Neil (1996) observed reduced preweaning mortality in sows that were fed on an ad libitum basis 4 d prior to farrowing compared with sows restricted fed until day 3 postpartum. The improved survival in the current study may be attributed to increased pig birth weight, improved milk output resulting in increased weaning weights, or a combination of multiple factors. Piglet characteristics related to survival are often interrelated, thus it is difficult to determine a single variable responsible for improved piglet survival (Baxter et al., 2008; Theil et al., 2014b). However, these results suggest feeding strategy prior to parturition may have an impact in the sows’ ability to raise her piglets to weaning.

Colostrum intake and yield were similar, indicating that feed amount or timing did not impact colostrum production in the short period prior to farrowing. In contrast, Mallmann et al. (2019) observed a decrease in colostrum yield when gilts were fed increasing amounts of gestation feed starting on day 90 of gestation. The authors concluded that the restricted fed gilts (1.8 kg/d) were mobilizing body protein and BF to meet demands for fetal growth and colostrum production prior to farrowing, with fat mobilization being prioritized for colostrum yield, therefore allowing for greater colostrum production in restricted-fed gilts. Because sow nutrient intake was not restricted to the severity as studied by Mallmann et al. (2019), sows in the current study likely mobilized minimal body protein and lipid stores to meet the colostrum demands if they were below the peripartum requirements as suggested by Dourmad et al. (2008). Decaluwe et al. (2014) observed decreased colostrum protein but increased colostrum lactose in sows fed increased feed on d 106 of gestation (4.5 vs. 1.5 kg/d), with no difference in colostrum IgG or IgA concentrations. Although not measured in the study, colostrum quality may have been altered due to differences in nutrient supply pre-farrow.

### Sow BW and BF Change

Lactation BW and BF loss was reduced when sows had consumed lactation feed ad libitum from entry to farrowing compared with sows fed four meals daily of 0.68 kg. Reduced BW and BF loss has also been observed in several studies where sows began ad libitum feed intake the last few days prior to farrowing (Neil, 1996; Cools et al., 2014; Decaluwe et al., 2014). The reduction in BF and BW loss is likely a result of increased feed consumption during the peripartum period (9.7 vs. 7.9 kg) and improved feed intake during the lactation period when sows were fed on an ad libitum basis. It is important to note that ad libitum feed intake prior to farrowing did not exceed 7 d. In previous studies that evaluated ad libitum or increased feeding strategies starting on d 90 of gestation, excess BW and BF gain occurred which contributed to decreased feed intake and increased sow body lipid mobilization in lactation (Weldon et al., 1994; Goncalves et al., 2016; Mallmann et al., 2019). These results suggest that feeding duration of a high energy and lysine diet may be largely responsible for the differences in sow body store mobilization and feed intake, and perhaps a short feeding duration with high feed intake peripartum is in fact beneficial to lactation performance.

### Sow Feed Intake

Lactation feed intake increased numerically in the present study in sows that were fed on an ad libitum basis compared with those restricted to one meal daily. Similarly, Decaluwe et al. (2014) observed a tendency for increased feed intake from day 108 of gestation through weaning in sows that had been fed three 1.5 kg meals/d prior to parturition compared with those receiving 1.5 kg daily. This change in feed intake could be due to a change in feeding behavior by allowing sows to determine their feed intake prior to farrowing, rather than restricted feeding. Cools et al. (2014) observed that peripartum voluntary feed intake was almost twice as much in sows fed on an ad libitum basis compared with sows restricted fed. Moreover, ad libitum feed allowance introduced 4 d prior to farrowing improved total lactation feed intake compared with those restricted until farrowing or 5 d after farrowing (Neil, 1996), and minimized BW and BF loss compared with sows restricted fed until 5 d after farrowing. These studies would be in contrast to results from an earlier study where primiparous sows were fed on an ad libitum basis from day 60 of gestation and had reduced lactation feed intake compared with sows that had been restricted fed in gestation (Weldon et al., 1994). Increased feed intake for an extended period of time can alter insulin sensitivity leading to suppressed lactation feed intake (Weldon et al., 1994). This further supports the idea that length of time that sows are offered ad libitum feed intake prior to farrowing can dictate the results of lactation feed intake.

### Litter Performance

The increase in average pig birth weight when sows were fed 0.68 kg every 6 h in the present study was unexpected. Because the feeding amount was the same in sows fed 0.68 kg every 6 h and fed 2.7 kg once daily, the increase in pig birth weight is not from an increase of total nutrients offered each day, but could be a factor of numerically lower litter size (15.7 vs. 16.0 or 16.1 total born) or changes in blood glucose. Several studies observed no change in litter weight or average pig birth weight when feeding sows ad libitum 4 to 7 d prior to farrowing (Neil, 1996; Cools et al., 2014; Decaluwe et al., 2014). In contrast, supplying gilts with 40 g standardized ileal digestible lysine and 13.3 Mcal ME from day 107 or 113 of gestation until farrowing increased piglet birth weight in gilts (Gourley et al., 2019). These different results may be attributed to the difference in parity, where older parity sows do not appear to partition additional energy consumed toward fetal growth (NRC, 2012). Nevertheless, additional research should be conducted to confirm if the number of meals offered pre-farrowing will improve performance compared with a single meal offering the same total daily nutrients.

The improvement in weaning weights observed from sows fed on an ad libitum basis prior to farrowing could have been a result of numerically higher lactation feed intake increasing milk production. As lactation feed intake is increased, milk output and litter growth increase (Hansen, 2012; Strathe et al., 2017). In the current study, it may be suggested that sows consuming feed ad libitum prior to farrowing were pre-conditioned to eat larger meals and transitioned more rapidly to consuming feed ad libitum in lactation compared with sows restricted fed prior to farrowing. In support of this, Guillemet et al. (2010) observed a quicker transition to lactation diets when sows were fed a high fiber (12.8% crude fiber) during gestation compared with a low fiber diet (3.5% crude fiber). The increase in lactation diet intake may be a factor of already consuming a greater amount of feed during gestation to maintain similar net energy to sows with low fiber diets. Therefore, feed intake in the last few days prior to parturition may not have an impact on birth weight, but rather is beneficial for establishing a level of feed intake that supports increased milk production and concomitantly litter growth throughout lactation.

### Sow Blood Glucose

The range in sow blood glucose at the onset of farrowing was similar to previous studies (Le Cozler et al., 1999; Feyera et al., 2018). The increase in sow blood glucose as farrowing duration increased suggests that sows will begin gluconeogenesis to support the uterine demands for energy near the end of parturition. Le Cozler et al. (1999) observed sow arterial glucose concentration remained constant as time since birth of first piglet increased, and rapidly increased after the birth of the last pig until time of placenta expulsion. This may be due an increased demand of glucose as a precursor for lactose in milk synthesis once parturition is complete (Farmer et al., 2015), or circulating hormones during parturition altering glucose regulation. Feyera et al. (2018) demonstrated glucose and triglyceride extraction by the uterus increased 1.4- and 6-fold, respectively, during parturition compared with after parturition. This may explain the rapid increase in arterial glucose following parturition, due to a decreased demand by the gravid uterus after the last pig is expelled.

The differences in blood glucose between treatments at all three time points are consistent, where sows fed on an ad libitum basis prior to farrowing had the highest blood glucose and sows fed 0.68 kg every 6 h had the lowest blood glucose. Similar to humans, it appears that increased frequency of meals may have reduced circulating blood glucose levels due to improved glucose tolerance (Carlson et al., 2007). This may benefit lactation feed intake as evidenced by a numerical improvement in feed intake observed in sows fed four daily meals compared with sows fed a single meal prior to farrowing. Long term, reduced glucose tolerance in late gestation may be a sign of gestational diabetes as reported by Kemp et al. (1996). However, due to differences in farrowing timing in relation to a consumed meal, it is unknown whether a sow was fasted prior to the start of farrowing, which may have increased the variability in initial glucose concentration we measured. Furthermore, Kemp et al. (1996) observed a weak correlation between sows with decreased fasting blood glucose on day 104 and increased piglet birth weights. This is in contrast to data suggesting that high blood glucose typically results in heavier birth weights (Vambergue and Fajardy, 2011). These conflicting results demonstrate more understanding of blood glucose effects on birth weight are needed in this area in swine.

### Subsequent Reproductive Performance

No change in subsequent reproductive or litter characteristics were observed regardless of feeding strategy prior to farrowing. It has been observed that if sows mobilize >12% of body protein during lactation, there will be reduced embryo survival (Vinsky et al., 2006) and decreased subsequent farrowing rate (Clowes et al., 2003). Although there were differences in sow BW change (*µ* = −4.0%) and BF change (*µ* = −14.8%) during lactation, it appears that they were not great enough to elicit a negative effect in subsequent reproductive performance. In the present study, a limited number of first parity sows were used due to the parity distribution of the sow herd, therefore further research is needed to determine if these same results would occur in first parity sows. Previous research suggests first parity sows may have improved subsequent reproductive performance when fed increased lysine and energy prior to farrowing (Gourley et al., 2019).

### Implications

In summary, it is important to consider the length of time of ad libitum feeding prior to farrowing, where the benefits in lactation feed intake, reduction in BF loss and improvements in litter growth can be observed. Feeding sows ad libitum lactation diet for an average of 3 d prior to farrowing increased weaning weight compared with sows fed once daily prior to farrowing. Sows limit-fed four times daily prior to farrowing had increased weaned percentage compared to sows fed one meal daily prior to farrowing. With levels of sow productivity in this study, there was no evidence feeding strategies from entry to the farrowing house until parturition impacted farrowing duration, birth interval, or stillborn rate. As litter size continues to increase, nutritional or management strategies to help reduce farrowing duration and improve piglet survival to weaning should continue to be investigated.
